# Evaluation of the Wondfo G6PD/Hb Test for glucose-6-phosphate dehydrogenase deficiency: preliminary performance, matrix equivalence, and usability

**DOI:** 10.1186/s12936-025-05436-0

**Published:** 2025-07-01

**Authors:** Rebecca K. Green, Stephanie Zobrist, Gornpan Gornsawun, Paw Khu Moo, Laypaw Archasuksan, Huyen Nguyen, Huong Nguyen, Cindy S. Chu, Emily Gerth-Guyette, Podjanee Jittamala, François Nosten, Sampa Pal, Germana Bancone, Gonzalo J. Domingo

**Affiliations:** 1 PATH – Diagnostics, Seattle, WA USA; 2 Shoklo Malaria Research Unit, Mahidol-Oxford Tropical Medicine Research Unit, Faculty of Tropical Medicine, Mahidol University, Mae Sot, Thailand; 3 PATH –Vietnam Country Program, Hanoi, Vietnam; 4 Centre for Tropical Medicine and Global Health, Nuffield Department of Medicine, University of Oxford, Oxford, UK; 5 Mahidol-Oxford Tropical Medicine Research Unit, Faculty of Tropical Medicine, Mahidol University, Bangkok, Thailand

**Keywords:** *Plasmodium vivax*, Glucose-6-phosphate dehydrogenase (G6PD), Point-of-care testing, Radical cure, Primaquine, Tafenoquine

## Abstract

**Background:**

Current treatment guidelines for radical cure of *Plasmodium vivax* malaria recommend the use of 8-aminoquinolines, which can result in life-threatening complications in people with glucose-6-phsophate dehydrogenase (G6PD) deficiency. Testing for this condition is recommended prior to administering such drugs. The Wondfo G6PD/Hb Test (Guangzhou Wondfo Biotech Co., Ltd., China) is a novel, quantitative point-of-care (POC) G6PD test that may help decentralize testing, expanding access to safe treatment options.

**Methods:**

Two studies were conducted: a retrospective diagnostic accuracy study on frozen venous whole blood specimens and a prospective matrix equivalency study. First, 300 frozen specimens from Mae Sot, Thailand were tested from July–August 2022 using the Wondfo test in laboratory and simulated field conditions. Reference testing for G6PD and Hb (spectrophotometer [Pointe Scientific, USA] and HemoCue [HemoCue AB, Sweden], respectively) was completed in the laboratory. Usability was evaluated among 10 intended users. Next, 225 participants were enrolled into a prospective matrix equivalency study from March–May 2023 in Memphis, Tennessee, USA. The Wondfo test was conducted at the POC with fingerstick capillary blood, and Wondfo and HemoCue tests were completed on fresh venous (K_2_EDTA) blood within 12 h. Remaining specimens were shipped to PATH for repeat Wondfo and reference testing.

**Results:**

The Wondfo G6PD measurement showed strong correlation under both laboratory and field conditions (R^2^>0.9). The area under the curve was 1.00 for deficient (95% CI: 1.00–1.00) and 0.99 for intermediate individuals (95% CI: 0.99–1.00). Sensitivity was high (1.00) across all conditions and groups (lower bound of the 95% CI ≥0.85). Good correlation was observed in both capillary and fresh venous blood against the reference and each other (R^2^>0.75). McNemar’s test showed no significant differences in classification between venous and capillary specimens. The Wondfo test achieved 98.2% (95%CI: 95.5–99.5%) overall agreement. All usability participants successfully completed quality control and test procedures, rating the test system highly for ease of use.

**Conclusions:**

The Wondfo G6PD/Hb Test demonstrates good diagnostic performance using current manufacturer thresholds across various conditions and both venous and capillary specimens. Comparable performance in both specimen types supports matrix equivalence. Usability is acceptable for end users, though refinements were recommended.

**Supplementary Information:**

The online version contains supplementary material available at 10.1186/s12936-025-05436-0.

## Background

Malaria remains a significant global health challenge, with an estimated 249 million cases and 608,000 deaths reported in 2022 [1]. This burden is disproportionately high in Africa, which accounts for 94% of malaria cases and 95% of malaria deaths [1]. While *Plasmodium falciparum* is the most prevalent and deadly of the malaria parasites, *Plasmodium vivax* also poses a significant threat due to its ability to cause recurrent infections from the dormant liver stage parasite [2]. *Plasmodium vivax* is most common in Asia and Latin America, contributing significantly to the overall malaria burden and causing severe illness as well as economic disruption in affected regions [2].

A variety of options exist for the treatment and prevention of malaria, including drugs in the 8-aminoquinoline family such as primaquine and, more recently, tafenoquine. These drugs are the only ones capable of radical cure, preventing relapse and eliminating liver stage parasites in *P. vivax* infections. People deficient in the glucose-6-phosphate dehydrogenase (G6PD) enzyme, however, are particularly susceptible to adverse health outcomes from radical cure treatments, including acute haemolytic anaemia. Due to these risks, the World Health Organization (WHO) recommends that “the G6PD status of patients should be used to guide administration of either primaquine or tafenoquine for preventing relapse [3].” Specifically, for administration of tafenoquine as well as high dose primaquine (1 mg/kg/day for 7 days), individuals should have G6PD activity above the threshold corresponding to 70% of normal [3, 4].

To date, a few qualitative tests for G6PD deficiency are available, including the fluorescent spot test as well as some lateral flow rapid diagnostic tests (CareStart G6PD RDT, AccessBio, USA and BinaxNOW G6PD Test, Alere, USA). These qualitative tests, however, cannot distinguish between normal and intermediate activity in heterozygous females, limiting safe treatment access for half the population [5–7]. Other assays, such as genetic and quantitative assays as well as qualitative assays such as the fluorescent spot test, require laboratory equipment and facilities, making them ill-suited to point-of-care (POC) testing. To date, only one commercially available quantitative POC G6PD test, the STANDARD™ G6PD Test (SD Biosensor, Republic of Korea), has been WHO Prequalified and widely used to support administration of radical cure treatments in endemic countries at the POC [8–13]. The availability of additional POC G6PD testing platforms is needed to enable radical cure treatment and support elimination efforts [14–16].

To address this gap, Guangzhou Wondfo Biotech Co., Ltd. (China) has developed the Wondfo G6PD/Hb Test, a quantitative POC biosensor that utilizes an optical signal to detect a change in fluorescence caused by the G6PD enzymatic reaction. The test presents numeric outputs of G6PD activity in U/g Hb. The findings of two studies conducted with the Wondfo G6PD/Hb Test to evaluate preliminary diagnostic performance, specimen matrix equivalency, and product usability are presented herein.

## Methods

### Ethical considerations

Results are presented from two studies: (1) a retrospective performance evaluation on frozen specimens in Mae Sot, Thailand, that also included a usability component, and (2) a matrix equivalency study conducted in Memphis, Tennessee, USA, using both fingerstick capillary and fresh venous specimens. The Thailand study was reviewed and approved by The Ethics Committee of the Faculty of Tropical Medicine, Mahidol University (Ref. No. TMEC 20–070), the Oxford Tropical Research Ethics Committee (Ref. No. 566-20), and WCG IRB (Ref. No. 1300295). The US study was reviewed and approved by WCG IRB (Ref. No. 1329637). All study participants were adults and provided written informed consent.

### Retrospective analysis of frozen specimens

A retrospective sub-study was conducted using frozen specimens (N = 300) collected during a cross-sectional diagnostic accuracy study in Mae Sot, Thailand. The original prospective study was conducted between November 2021 through February 2022 and enrolled participants from Burmese and Karen migrant populations living along the Thailand-Myanmar border. All participants in the parent study were adults (≥18 years of age), able to speak and understand Karen or Burmese, and willing and able to provide informed consent and comply with study requirements. Individuals with severe malaria, other severe illness, or self-reportedly having received a blood transfusion in the last three months were excluded. An enriched population with known abnormal G6PD status established through previous studies (N = 16, 5.3% of the sample) was used to supplement standard recruitment, ensuring a range of G6PD activity was tested. Venous blood samples collected from these individuals were directly stored in liquid nitrogen until analysed retrospectively during this study from July to August 2022.

Specimens were thawed on ice and mixed thoroughly prior to testing. All index (Wondfo G6PD/Hb Test) and reference (quantitative spectrophotometry and HemoCue) testing was completed within 20 min of thawing. The Wondfo test was run in duplicate under two different environmental conditions: temperature and humidity-controlled laboratory conditions vs. temperature and humidity uncontrolled simulated field conditions. All test operators were blinded to any prior known G6PD status associated with the specimens. Operators of the investigational Wondfo test were blinded to the results of the G6PD reference assay and vice versa.

### US prospective matrix equivalency study

A prospective cross-sectional diagnostic accuracy study was conducted in Memphis, Tennessee, USA. Eligible participants were healthy blood donors, ≥18 years of age, self-reported Black/African American, afebrile, and able to provide written informed consent. Individuals who self-reported having received a blood transfusion in the last three months were excluded. Participants (n = 225) were enrolled from March to May 2023. Following completion of informed consent, the Wondfo G6PD/Hb Test was used at the POC on capillary blood collected by fingerstick. Venous blood (2mL) was subsequently collected into a K_2_EDTA tube and stored at 4°C until ready for further testing. Samples were brought to room temperature, and on site laboratory testing of the Wondfo and HemoCue tests were completed using the fresh venous blood within 12 h of collection. Specimens were then shipped on ice overnight to the PATH laboratory (Seattle, WA, USA), where they were brought to room temperature for testing on the reference spectrophotometer, repeat Wondfo, and HemoCue within 72 h of collection.

### Testing methods

#### Wondfo G6PD/Hb Test

The Wondfo G6PD/Hb Test was conducted in both studies in accordance with the manufacturer’s instructions for use and applying the proposed thresholds following data collection to classify normal, intermediate, and deficient individuals as described in Table 1. The suitability of the proposed thresholds was evaluated through the matrix equivalency study and is discussed later.

**Table 1:** Proposed manufacturer thresholds for classifying G6PD activity measured by the Wondfo G6PD/Hb Test, as described in the instructions for use

Males		Females	
G6PD Deficient	< 3 U/g Hb	G6PD Deficient	< 3 U/g Hb
G6PD Normal	≥ 3 U/g Hb	G6PD Intermediate	3–6.99 U/g Hb
		G6PD Normal	≥ 7 U/g Hb

Prior to allowing any samples to be tested, the analyzer requires an internal calibration and quality control procedure to be completed. This must be completed using the QC cuvette every 24 h or when prompted by the analyzer (e.g., if environmental conditions change substantially). This internal quality control procedure was completed accordingly in both studies.

**Fig. 1:**
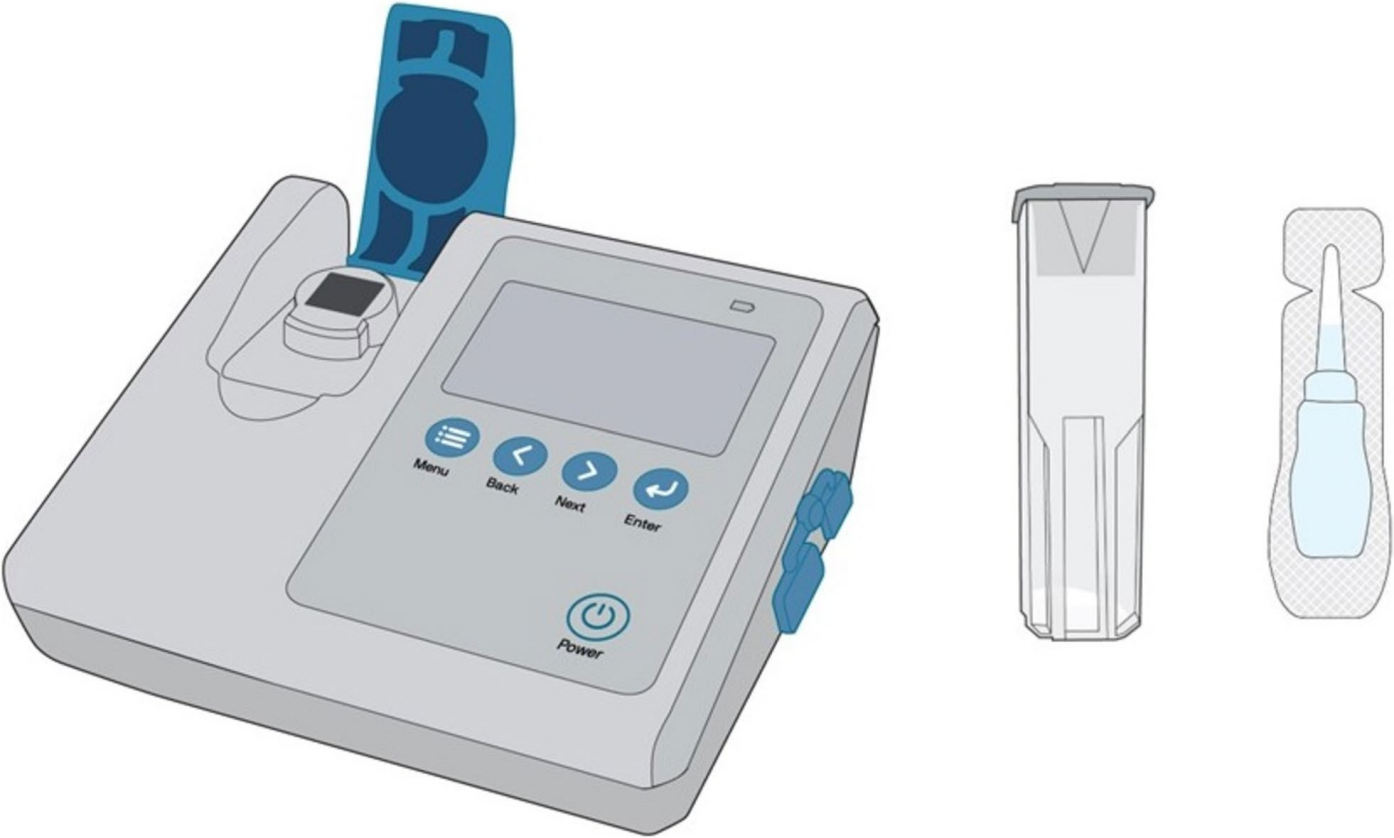
Wondfo G6PD/Hb Test Analyzer, cuvette with lyophilized reagent, and buffer tube

The test uses an optical signal to measure G6PD activity and haemoglobin concentration. The G6PD enzyme in the specimen converts NADP+ to NADPH in the prepared cuvette. The resulting change in absorbance over time is measured by the analyzer to capture the enzyme activity. The enzyme activity is normalized for haemoglobin concentration, also measured from the same cuvette to account for the red blood cell content in the cuvette. To perform the test, the test cuvette containing lyophilized reagent is prepared by adding one tube of buffer. Then, 10 µL of capillary or venous whole blood is added and the cuvette is gently inverted several times to mix. The cuvette is inserted into the analyzer and the test is started by pressing the “enter” button. The test takes approximately 5 minutes to run, and the result is displayed on the screen. The analyzer weighs approximately 490g and measures 128 x 142 x 63 mm; the test components are shown in Fig. 1 [16]. See Supplemental File 1 for a summary of the complete test procedure.

#### HemoCue

Haemoglobin was determined using the HemoCue 301+ for the Thailand study and the 201+ for the US study (HemoCue AB, Ängelholm, Sweden). The machines used in the US study underwent regular quality control testing using Hi and Lo control material twice per week, and the machines used in Thailand were maintained per local laboratory practices, though did not undergo regular quality control testing for study purposes. For both studies, the HemoCue test was run at the same time as the reference G6PD assay and used to normalize the reference G6PD result.

#### G6PD reference assay

Both studies employed automated quantitative spectrophotometry (Shimadzu Corporation, Kyoto, Japan) as the G6PD reference assay using the Pointe Scientific G6PD reagent kit (Pointe Scientific, Canton, MI; Cat No. G7583) according to the manufacturer’s instructions. The assay was run in duplicate and mean enzyme activity determined using temperature correction at 30°C. Samples in Thailand were retested if the coefficient of variation between two measurements exceeded 10%. The maximum variation observed between readings was 61% in deficient specimens (due to the low activity) and 9.7% in normal specimens. Samples in the US study were retested if the absolute difference between readings exceeded 2 U/g Hb, and the maximum difference observed was 0.87 U/g Hb. The rate of change in absorbance at 340 nm over 5 min was measured and G6PD activity calculated in U/g Hb. Normal, intermediate, and deficient controls from Analytical Control Systems, Inc. (Fishers, IN; Cat. Nos. HC-108, HC-108IN, and HC-108DE, respectively) were run using the same method on each day of testing. G6PD values were corrected according to the manufacturer’s instructions by applying a temperature correction factor (1.37) at 30°C.

In Thailand, reference testing was completed in the site’s haematology laboratory within 20 min of thawing. In the US study, reference testing was completed at the PATH laboratory within 72 h of specimen collection.

### Usability evaluation

A formative usability evaluation was conducted with intended end users of the Wondfo G6PD/Hb Test in Thailand. The objectives of this evaluation were to understand 1) how well users moved through the workflow of the test and internal quality control (QC) procedures, 2) how well users could read, record, and interpret the test results, and 3) identify critical changes needed to the product and Instructions for Use (IFU) ahead of clinical studies.

Following completion of informed consent, participants were given an adapted version of the IFU, which was translated into the local language, and brief training on the test procedure which included a product demonstration. Participants were then asked to complete the internal QC and test procedure using imitation blood, employing the “think aloud” method. User-test interactions were observed by the moderator and quantitative data was collected using an objective metric sheet. Participants were then asked to interpret the results of eight example test result outputs (2 deficient, 2 intermediate, 2 normal, 1 out of measuring range, 1 error) and classify them as normal, intermediate, deficient, or error/out of measuring range according to the IFU. A semi-structured exit interview was also conducted to collect feedback on the user’s experience with the test and accompanying IFU. This included a standard Systems Usability Scale (SUS) [17].

### Analysis

#### Reference G6PD activity determination

G6PD activity was normalized by haemoglobin concentration derived from the respective reference HemoCue test and is reported as U/g Hb. Absolute G6PD values on the reference assay were normalized to facilitate inter-laboratory comparison, according to standard methods [4, 18, 19]. Reference values were expressed as the percentage of each site’s adjusted male median determined from 36 randomly selected males with normal G6PD activity within the G6PD reference range for the reference assay in all males. These were subsequently excluded from the analysis in Thailand but were retained in the Memphis analysis due to sample size constraints. Quantitative thresholds for G6PD activity categories at 30% (deficient males and females) and 70% (intermediate females) were calculated to assess performance.

#### Diagnostic performance and matrix equivalence

All analyses were conducted in R using the pROC, BlandAltmanLeh, epiR, and ggplot2 packages [20–23]. Linear regressions and Bland-Altman plots were constructed for the Wondfo G6PD/Hb Test against the reference measurements for each condition (e.g., laboratory, simulated field, venous, capillary). The same plots were constructed for capillary measurements against venous measurements to examine matrix equivalency. Respective R^2^ values were calculated from the equations. Receiver Operating Characteristic (ROC) Curves were similarly constructed for each condition at the 30% and 70% thresholds and the area under the curve was calculated. A threshold optimization analysis was completed using the pROC package to compute Youden’s J statistic, locating the optimal threshold at which sensitivity and specificity were maximized based on the ROC curves [20, 24].

Sensitivity, specificity, and overall percent agreement were calculated with 95% confidence intervals (CI) for each condition at the 30% and 70% thresholds. Two by two contingency tables were constructed for deficient and intermediate classifications between venous and capillary specimens (from the same individuals). The thresholds identified through the optimization analysis were then compared to the thresholds stated in the Wondfo G6PD/Hb Test IFU and applied to each data set to recalculate diagnostic performance characteristics. McNemar’s test for marginal homogeneity was conducted using the contingency tables to assess significant differences in test classification between venous and capillary specimens.

#### Product usability

Demographic characteristics of the usability study participants were summarized. Average task completion time was calculated in Excel and a descriptive task analysis was completed. SUS scores were computed using the standard method prior to calculating the median, mean, and range [17].

## Results

### Study population and setting

Between the two preclinical evaluations, N = 489 samples were evaluated (Table 2). Both populations had an average age in the mid- to upper thirties (34.5 and 39.3 years in Thailand and the US, respectively). The study population in Thailand was predominantly female, whereas the US population was predominantly male.

**Table 2:** Demographic information from Thailand and US studies

	Mae Sot, Thailand	Memphis, USA
N	264	225
Age (Mean (SD))	34.5 (11.1)	39.3 (12.1)
Sex = Male (%)	91 (34.5)	182 (80.9)
Moderate Anaemia* (%)	9 (3.4)	13 (5.8)
*Spectrophotometer G6PD Status (%)*		
Normal	207 (78.4)	199 (88.4)
Intermediate	23 (8.7)	6 (2.7)
Deficient	34 (12.9)	20 (8.9)

^*^Per WHO-established thresholds where 8.0–10.9 g/dL was considered moderate anaemia in this population [25]; no severe anaemia was observed in either population, so all remaining participants had no to mild anaemia

In the Thailand study, operating temperature and humidity under laboratory conditions averaged 25.6°C (22.8–27.9°C) and 40% (28–52%), respectively, as compared to 30.1°C (28.8–31.5°C) and 66% (58–71%) for simulated field conditions.

Both studies used ROC analysis to evaluate the test’s performance in identifying G6PD deficient and intermediate (female) cases. All computed areas under the curve from the ROC analysis were >0.9 (Table 3).

**Table 3:** Area under the curve (95% CI) across conditions and specimen types against reference spectrophotometer

Location	Specimen	Setting	Deficient (30% threshold, males & females)	Intermediate (70% threshold, females only)
Mae Sot, Thailand	Frozen venous	Laboratory	1.000 (1.000–1.000)	0.995 (0.989–1.000)
	Frozen venous	Simulated field	1.000 (1.000–1.000)	0.993 (0.984–1.000)
Memphis, USA	Fresh capillary	POC	0.996 (0.998–1.000)	0.931 (0.855–1.000)
	Fresh venous	Laboratory	0.995 (0.986–1.000)	0.976 (0.936–1.000)

Matrix equivalence and diagnostic performance

### Matrix equivalence and diagnostic performance

**Fig. 2:**
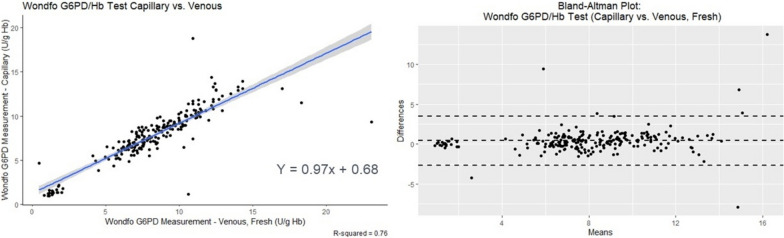
Linear Regression and Bland-Altman Plot for fresh capillary vs. fresh venous specimens

A good correlation (R^2^ = 0.76) was observed between the two specimen types in the US study, with the Bland-Altman Plot demonstrating a high degree of clustering around zero (Fig. 2). McNemar’s test for both deficient and intermediate classifications on the Wondfo test between specimen types yielded a p-value >0.5, indicating homogeneity in classification between specimen types.

Among the G6PD deficient cases there were two discordant samples between the venous and capillary specimens. Examination of the reference assay for these two samples showed that both were false deficient (one venous and one capillary) with normal G6PD activity (70% and 136%, respectively).

High sensitivity and specificity were observed using the Wondfo IFU thresholds across all specimen types and conditions for deficient individuals (reference G6PD activity <30%) (Table 4). For intermediate females (reference G6PD activity 30–70%), sensitivity was excellent (1.00) for the frozen samples (Table 4). The fresh specimens showed good sensitivity for intermediate females, though with wide 95% confidence intervals, 100% (95% CI: 54–100%) and 83% (95% CI: 34–99%) in fresh capillary and venous specimens, respectively. All misclassified females had reference G6PD activity at or above 70%. Specificity for intermediate females was low when testing frozen venous specimens under both laboratory and simulated field conditions (37%, 95% CI: 30–45% and 34%, 95% CI: 26–42%, respectively) but increased in both fresh capillary and venous specimens (89%, 95% CI: 74–97% and 92%, 95% CI: 78–98%, respectively) (Table 4). Overall percent agreement was poor on the frozen specimens under both conditions but excellent on both fresh specimen types (Table 5). Three-by-three agreement for each condition and specimen type is included in Supplemental Tables 1-4. Regressions and Bland-Altman Plots for all specimen types can be found in Supplemental Figs. 1 and 2.

**Table 4:** Diagnostic performance (95% CI) across conditions and specimen types using Wondfo thresholds against reference spectrophotometer

Location	Specimen	Setting	Deficient (30% threshold, males & females)		Intermediate (70% threshold, females only)	
			Sensitivity	Specificity	Sensitivity	Specificity
Mae Sot, Thailand	Frozen venous	Laboratory	1.00 (0.90–1.00)	0.94 (0.90–0.97)	1.00 (0.85–1.00)	0.37 (0.30–0.45)
	Frozen venous	Simulated field	1.00 (0.89–1.00)	0.94 (0.90–0.96)	1.00 (0.85–1.00)	0.34 (0.26–0.42)
Memphis, USA	Fresh capillary	POC	1.00 (0.83–1.00)	0.99 (0.97–1.00)	1.00 (0.54–1.00)	0.89 (0.74–0.97)
	Fresh venous	Laboratory	1.00 (0.83–1.00)	0.99 (0.97–1.00)	0.83 (0.34–0.99)	0.92 (0.78–0.98)

**Table 5:** Overall percent agreement (95% CI) across conditions and specimen types using Wondfo thresholds against reference spectrophotometer

Location	Specimen	Setting	Overall percent agreement (95% CI)
Mae Sot, Thailand	Frozen venous	Laboratory	59.5% (53.3–65.4%)
	Frozen venous	Simulated field	57.4% (51.4–63.6%)
Memphis, USA	Fresh capillary	POC	98.2% (95.5–99.5%)
	Fresh venous	Laboratory	97.8% (94.9–99.3%)

### Threshold analysis

Given the drastically different performance in intermediate females on the frozen samples in Thailand, a threshold optimization analysis was conducted separately on each data set from Thailand and the US (Table 6). Applying the optimized thresholds to the Thailand data yielded 94% (95% CI: 89–97%) intermediate female specificity under both laboratory and simulated field conditions as well as 100% (95% CI: 98–100%) deficient specificity under both conditions. Applying the optimized thresholds to the US data did not change diagnostic performance in either specimen type.

**Table 6:** Threshold optimization from ROC curves

Source of determination	Threshold
*Wondfo IFU*	
Deficient	<3.0 U/g Hb
Intermediate (females only)	≥3.0, <7.0 U/g Hb
*Optimized (Thailand)*	
Deficient	<0.64 U/g Hb
Intermediate (females only)	≥0.64, <4.66 U/g Hb
*Optimized (US, capillary)*	
Deficient	<3.00 U/g Hb
Intermediate (females only)	≥3.00, <6.74 U/g Hb
*Optimized (US, venous)*	
Deficient	<3.07 U/g Hb
Intermediate (females only)	≥3.07, <7.41 U/g Hb

### Product usability

This study included ten participants in total, ranging in age from 19 to 53 (median age 27.5); most of the participants were female (70%). Of the participants, three were laboratory technicians, two were health workers at the Shoklo Malaria Research Unit clinic, and the remaining five were community engagement staff. Participants had a range of professional experience with 40% having <1 year and 30% having >10 years; job responsibilities primarily included sample collection (e.g., blood), patient consultation/treatment, and laboratory analyses. Over half of the participants (60%) had previously used a G6PD Test, namely the STANDARD G6PD Test and the fluorescent spot test.

The average completion time for the internal QC and test procedure was 8.5 and 11.5 min, respectively. Most participants were able to complete both procedures smoothly, following a demonstration. Challenges were noted in navigating to the correct interface and mixing/transferring the sample. All participants correctly recorded the G6PD and Hb results from all example result screens. Interpretation errors were infrequent (8/80, 10%) but were more common with deficient and intermediate results. Of the interpretation errors, two were deficient misclassified as normal (10% error rate for deficient results), two were intermediate misclassified as deficient, and two were intermediate misclassified as normal (20% error rate for intermediate results, though only 10% of these could result in an adverse clinical outcome). The out of measuring range (Hi) result was misclassified as deficient and normal, though the latter would be an acceptable interpretation in clinical practice. Overall, 5 of these 8 errors (62.5%) could result in adverse clinical outcomes.

The median composite SUS was 70 (mean 66.75, range 45–85), indicating good usability overall; only two participants had a SUS score in the “OK” range and none rated the test poor or below [26]. All participants felt that the IFU was clear and easy to understand. Participants liked the portability of the analyzer, the symbols included on the analyzer display, the short procedure time, and the quantitative result. Participants disliked the number of things on the analyzer display and that it switched to standby mode too quickly. All participants’ recommendations for the test procedure and component improvements were related to the cuvette, including making the lid easier to open, providing clearer instructions about how to hold it, and making it more apparent how it should be inserted in the analyzer. In response to these suggestions, the manufacturer made the tab on the cuvette lid longer, created a cuvette holder, and improved the test instructions for use prior to the clinical validation studies.

## Discussion

POC G6PD tests are critical to enable radical cure of *P. vivax* malaria. At present, only one commercially available quantitative product, the STANDARD G6PD test (SD Biosensor, Republic of Korea), has been widely used in the context of malaria case management. Quantitative tests allow the differentiation between deficient, intermediate, and normal G6PD activity levels at the POC, to ensure women have equal access to this curative treatment [7]. The WHO guidelines for malaria recommend use of a semi-quantitative or quantitative test to inform treatment with single dose tafenoquine or the high dose regimen of primaquine at 1 mg/kg/day for 7 days [3]. These studies were conducted to evaluate the preliminary diagnostic performance and usability of an additional quantitative POC G6PD test—the Wondfo G6PD/Hb Test—to inform further product development needs in advance of clinical validation studies.

The test demonstrated high overall diagnostic performance under both laboratory and simulated field conditions as well as in fresh venous and capillary specimens. Comparable performance across specimen types (R^2^ = 0.76) supports using the test with capillary or venous specimens. Consistent test performance under varied temperature and humidity conditions indicates the test is robust enough for the intended context of use supporting malaria case management in endemic settings.

The low specificity observed in testing frozen intermediate specimens was likely due to a small but significant decrease in G6PD activity in this specimen type, which has been observed in similar studies analyzing frozen specimens, perhaps due to the Wondfo test’s simplified procedure rendering it more sensitive to degraded samples or interfering substances [27, 28]. This is further illustrated in the optimized thresholds, which are exceedingly low in the Thailand study (where frozen specimens were tested) compared to the US study (where fresh specimens were tested). The optimized thresholds in the Thailand study are impractically low, as a cutoff using such a small portion of the testing range will be more prone to missing at-risk individuals, underscoring this anomaly in performance as a specimen-related phenomenon rather than a problem with the test itself. Further, frozen specimens are not a claimed specimen type for this product.

The imprecision noted in the sensitivity of intermediate females for fresh capillary and venous specimens is likely due to the low number of females enrolled in the study (<20% of the study population). Note that a comparable level of imprecision in this point estimate was not observed in the Thailand study, in which approximately 65% of the study population was female. Despite the low number of females in the US study (with fresh samples), performance remained good, and the optimized thresholds were almost identical to the IFU thresholds. Further, all six misclassified females had greater than or equal to 70% G6PD activity per reference testing. The one individual classified as intermediate on reference testing (reference G6PD activity = 70%) was classified as intermediate on capillary testing but normal on venous testing according to the stated manufacturer thresholds. Of the five other individuals classified as normal per reference testing, two were classified as intermediate on both venous and capillary testing, one was classified as intermediate on capillary testing but normal on venous testing, and one was classified as normal on capillary testing but intermediate on venous testing. The remaining normal individual was classified as deficient on capillary testing and normal on venous testing, where the measured haemoglobin per the Wondfo device was almost 2 g/dL higher in the capillary sample compared to the venous sample (which was 0.5 g/dL lower than the corresponding reference measurement). From a clinical perspective, four of these five individuals who should be eligible for tafenoquine would not be eligible under the current manufacturer thresholds. While minimizing the risk of acute haemolytic anaemia, this would place these individuals at greater risk by receiving a less effective radical cure treatment that may increase the likelihood of malaria relapses and the associated risks of increased morbidity and population transmission. It will be critical to balance these risks as the test moves towards market entry.

The Wondfo G6PD/Hb Test was well-liked by users, with a median score of 70 on the SUS and very few user errors. The interpretation errors observed for intermediate results may be related to the test having different thresholds than that of the only other widely used POC quantitative test (the STANDARD^TM^ G6PD Test), with which most participants had prior experience. High usability and user satisfaction are crucial to the success of a product, and these findings suggest the Wondfo G6PD/Hb Test is easy to use. Suggestions from users to improve the product, such as making the lid easier to open and including a cuvette holder to make it easier to handle, have been incorporated into the product design, further improving the user experience and increasing the probability of future product uptake.

In this preliminary study, Wondfo G6PD/Hb Test demonstrates promising diagnostic performance to support malaria case management. Further product development efforts, including prospective clinical evaluations in the intended context of use, are warranted. Product usability should also be re-assessed following enhancements made to the test and its instructional materials.

## Limitations

As reference haemoglobin concentration was measured by the HemoCue device rather than the gold standard haematology analyzer, the performance of the Wondfo G6PD/Hb Test in the measurement of haemoglobin was not assessed. While the use of frozen specimens has limitations, it was deemed an appropriate approach for this preliminary assessment to avert unnecessary blood sampling and prioritize efficiency in the product development process prior to investment in large scale clinical validation studies. Future prospective clinical studies should evaluate the performance of the product in the intended use setting.

## Conclusions

The Wondfo G6PD/Hb Test is excellent for identifying G6PD deficient individuals and good for identifying G6PD intermediate individuals. Overall, the test is fit-for-purpose to meet the needs of healthcare staff involved in malaria case management and shows robust performance in varied environmental conditions. The test shows good specimen equivalence on its two indicated specimen types and the usability is considered acceptable by target end users.

## Supplementary Information

Additional file1

Additional file2

Additional file3

Additional file4

## Data Availability

The datasets generated and/or analyzed during the current study are available in the Harvard Dataverse (https://doi.org/10.7910/DVN/JRUCV9).

## Abbreviations

CI: Confidence interval
G6PD: Glucose-6-phosphate dehydrogenase
Hb: Haemoglobin
IFU: Instructions for Use
IRB: Institutional review board
K_2_EDTA: Dipotassium ethylene diamine tetraacetic acid
POC: Point-of-care
QC: Quality control
SUS: Systems usability scale
WHO: World Health Organization
