# Supplementary figures and images for: Evaluation of the Wondfo G6PD/Hb Test for glucose-6-phosphate dehydrogenase deficiency: preliminary performance, matrix equivalence, and usability
Source: Malar J. 2025 Jul 1;24:201. doi: 10.1186/s12936-025-05436-0 (PMC12210538; doi:10.1186/s12936-025-05436-0)

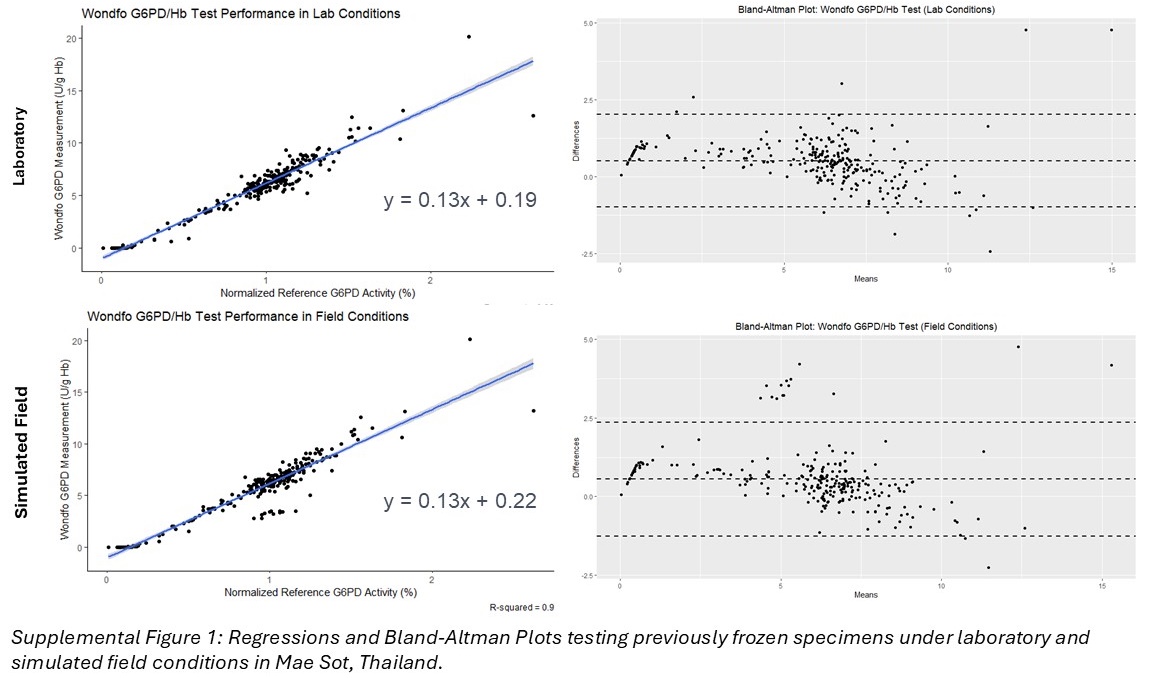

Supplement: Supplementary file 1 — Additional file1 [file 12936_2025_5436_MOESM1_ESM.jpg]

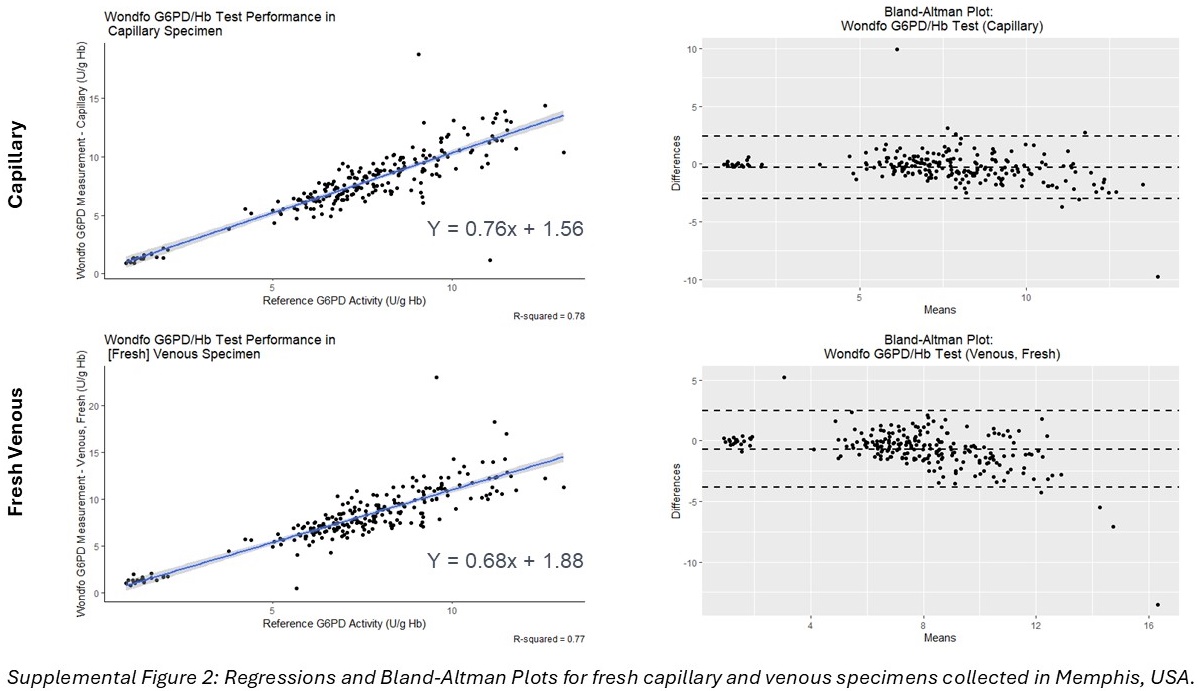

Supplement: Supplementary file 2 — Additional file2 [file 12936_2025_5436_MOESM2_ESM.jpg]
