# Supplementary material for: Evaluation of the Wondfo G6PD/Hb Test for glucose-6-phosphate dehydrogenase deficiency: preliminary performance, matrix equivalence, and usability
Source: Malar J. 2025 Jul 1;24:201. doi: 10.1186/s12936-025-05436-0 (PMC12210538; doi:10.1186/s12936-025-05436-0)
Supplement: Supplementary file 3 — Additional file3 [file 12936_2025_5436_MOESM3_ESM.docx]

| 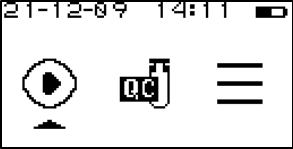 | 1. Turn on the analyzer and enter the testing interface. |
| --- | --- |
| 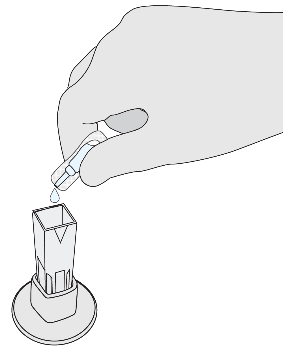 | 1. Open the cuvette and empty buffer solution into it. |
| 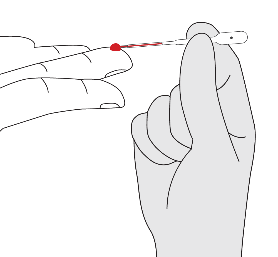 | 1. Collect specimen. |
| 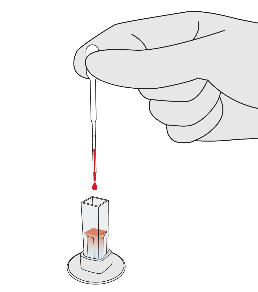 | 1. Add blood specimen to the cuvette. |
| 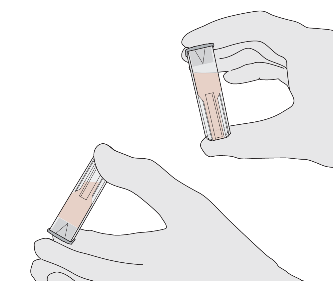 | 1. Invert 10-20 times to mix thoroughly. |
| 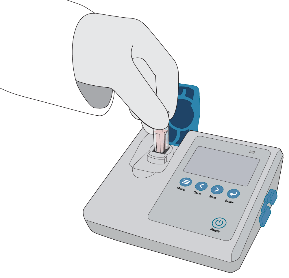 | 1. Insert cuvette into the sample well. |
| 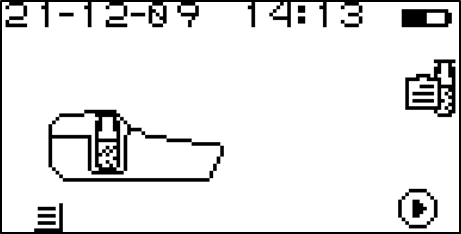 | 1. Start the test. |
