# Supplementary material for: Evaluation of the Wondfo G6PD/Hb Test for glucose-6-phosphate dehydrogenase deficiency: preliminary performance, matrix equivalence, and usability
Source: Malar J. 2025 Jul 1;24:201. doi: 10.1186/s12936-025-05436-0 (PMC12210538; doi:10.1186/s12936-025-05436-0)
Supplement: Supplementary file 4 — Additional file4 [file 12936_2025_5436_MOESM4_ESM.docx]

Supplemental Table 1. 3x3 agreement under laboratory conditions using proposed thresholds.

|  | | **Spectrophotometer (reference)** | | | |
| --- | --- | --- | --- | --- | --- |
|  |  | Normal | Intermediate | Deficient | *Sum* |
| **Wondfo G6PD/Hb Test** | Normal | 112 | 0 | 0 | *112* |
|  | Intermediate | 93 | 11 | 0 | *104* |
|  | Deficient | 2 | 12 | 34 | *48* |
|  | *Sum* | *207* | *23* | *34* | *264* |

Supplemental Table 2. 3x3 agreement under simulated field conditions using proposed thresholds.

|  | | **Spectrophotometer (reference)** | | | |
| --- | --- | --- | --- | --- | --- |
|  |  | Normal | Intermediate | Deficient | *Sum* |
| **Wondfo G6PD/Hb Test** | Normal | 106 | 0 | 0 | *106* |
|  | Intermediate | 97 | 12 | 0 | *109* |
|  | Deficient | 4 | 11 | 33 | *48* |
|  | *Sum* | *207* | *23* | *33* | *263* |

Supplemental Table 3. 3x3 agreement for fresh capillary specimens using proposed thresholds.

|  | | **Spectrophotometer (reference)** | | | |
| --- | --- | --- | --- | --- | --- |
|  |  | Normal | Intermediate | Deficient | *Sum* |
| **Wondfo G6PD/Hb Test (Capillary)** | Normal | 195 | 0 | 0 | *195* |
|  | Intermediate | 3 | 6 | 0 | *9* |
|  | Deficient | 1 | 0 | 20 | *21* |
|  | *Sum* | *199* | *6* | *20* | *225* |

Supplemental Table 4. 3x3 agreement for fresh venous specimens using proposed thresholds.

|  | | **Spectrophotometer (reference)** | | | |
| --- | --- | --- | --- | --- | --- |
|  |  | Normal | Intermediate | Deficient | *Sum* |
| **Wondfo G6PD/Hb Test (Venous)** | Normal | 195 | 1 | 0 | *196* |
|  | Intermediate | 3 | 5 | 0 | *8* |
|  | Deficient | 1 | 0 | 20 | *21* |
|  | *Sum* | *199* | *6* | *20* | *225* |
